# The TcVps34–TcVps15 complex regulates parasite metacyclogenesis and host cell infection in *Trypanosoma cruzi*

**DOI:** 10.3389/fcimb.2026.1668322

**Published:** 2026-04-21

**Authors:** Alejandra Cecilia Schoijet, Joaquín Iolster, Guillermo Daniel Alonso

**Affiliations:** 1 Signaling and Adaptive Mechanisms in Trypanosomatids Laboratory, Instituto de Investigaciones en Ingeniería Genética y Biología Molecular “Dr. Héctor N. Torres”, Consejo Nacional de Investigaciones Científicas y Técnicas, Buenos Aires, Argentina; 2 Departamento de Química Biológica, Facultad de Ciencias Exactas y Naturales, Universidad de Buenos Aires, Buenos Aires, Argentina; 3 Departamento de Fisiología, Biología Molecular y Celular, Facultad de Ciencias Exactas y Naturales, Universidad de Buenos Aires, Buenos Aires, Argentina

**Keywords:** autophagy regulation, cellular infection, Chagas´ disease, metacyclogenesis, *Trypanosoma cruzi*

## Abstract

The processes of autophagy and metacyclogenesis are essential for the survival and progression of the cell cycle of *Trypanosoma cruzi*, the etiological agent of Chagas disease. There is experimental evidence of an interconnection between both pathways in this parasite. Previously, it has been shown that the phosphatidylinositol 3-kinase TcVps34 and its regulatory protein kinase, TcVps15, are essential for autophagy in *T. cruzi*. In this work, we studied the role of these two proteins in metacyclogenesis and infection. Here, we demonstrate that both proteins are involved in the differentiation process. In addition, the alternative overexpression of either TcVps15 or TcVps34 increases the autophagic process after the first two hours of induction of metacyclogenesis and increases the activity of degradative compartments. Finally, both overexpressing cell lines show reduced infection levels in Vero host cells; additionally, these parasites display a downregulation of the surface glycoprotein gp82—associated with promoting the host infection—and a mild upregulation of gp90, known to negatively modulate infectivity. In conclusion, this study highlights the critical roles of the TcVps34-Vps15 complex in regulating the interplay between autophagy and metacyclogenesis in *Trypanosoma cruzi*, suggesting a contribution of both proteins to a successful infection establishment.

## Introduction

The phosphatidylinositol 3-kinase (PI3K) family is a diverse group of lipid kinases, that have been found in all eukaryotic cell types examined to date, and are linked to an incredibly diverse set of key cellular functions, including cell growth, proliferation, motility, differentiation, survival, and intracellular trafficking. Vps34 is the unique member of the class III PI3K family, which forms a complex with the Vps15 kinase and Beclin 1, and depending on the association of other proteins, it forms the complex I in eukaryotes, involved in autophagy and the complex II, involved in endosomal trafficking (Ohashi, 2021).

*Trypanosoma cruzi* is a protozoan parasite that causes Chagas disease. It exhibits a widespread distribution worldwide, with the largest number of cases being observed in Latin American countries, but with significant prevalence rates being reported in non-endemic countries in North America and Europe (Gómez-Ochoa et al., 2022). It is estimated that around 7 million people are infected with *T. cruzi* worldwide and 70 million people are at risk of infection, leading to about 12,000 deaths annually, according to the World Health Organization (World Health Organization, 2023). As the parasite develops its life cycle and alternates between the mammalian host and the insect vector, it goes through numerous fluctuations in nutritional availability, osmolarity, pH, and temperature, among others. As epimastigotes multiply and colonize the gut of the insect vector, they undergo differentiation into metacyclic trypomastigotes under certain conditions, a process named metacyclogenesis. It has been demonstrated that starvation triggers this process in *T. cruzi* (Vanrell et al., 2017).

Autophagy is a crucial cellular process that degrades and recycles cellular components, and it plays an essential role in various biological processes, including development, differentiation, response to stress, and cellular homeostasis. Similar to other eukaryotic organisms, *T. cruzi* possesses a functional autophagy pathway that is activated in response to nutritional stress, although many proteins in the pathway remain to be characterized (Alvarez et al., 2008; Romano et al., 2023). Recently, it was shown that stimulation of autophagy promotes Cruzipain (the main cysteine protease enzyme produced by *T. cruzi*) delivery to reservosomes and increases its hydrolytic activity in these compartments, resulting in enzymatic activation and self-processing (Losinno et al., 2021). It is worth mentioning that Cruzipain has been implicated in evading host immune responses due to its ability to degrade host proteins, altering immune recognition (Saha et al., 2024). As mentioned above, the fact that autophagic mechanisms are involved in the differentiation from non-infective to infective forms (epimastigotes to metacyclic trypomastigotes), suggests that autophagy helps not only in survival but also facilitates transitions between different developmental stages. In addition, the autophagic process may contribute to how these parasites interact with host cells during invasion and infection. Some studies suggest that specific proteins involved in autophagy help these parasites to evade host immune responses or modulate signaling pathways conducive to their invasion (Casassa et al., 2019; Romano et al., 2023).

Previously in our laboratory, we identified the first PI3K in *T. cruzi*, named TcVps34, with similarity to its yeast counterpart, Vps34p. We have demonstrated that this enzyme plays a prominent role in vital processes for the parasite’s survival, such as osmoregulation, vesicular acidification, and vesicular trafficking (Schoijet et al., 2008). Also, we have characterized the serine-threonine protein kinase TcVps15 and confirmed that it forms a complex with TcVps34, and that both participate in autophagy in this parasite (Schoijet et al., 2017). It has also been shown more recently that TcBeclin1 co-localizes with TcVps34 and its overexpression plays an important role in the survival of *T. cruzi*, possibly exerting a role in autophagy (Nájera et al., 2024).

In this context, the interplay between autophagy and metacyclogenesis represents an interesting area of study within parasitology that highlights how *Trypanosoma cruzi* adapts morphologically under environmental pressures while optimizing its lifecycle for successful transmission between hosts.

In this work, we demonstrate that transgenic parasites overexpressing TcVps34 or TcVps15, which have elevated levels of autophagy, also show increased metacyclogenesis in *T. cruzi*. Furthermore, concerning their interaction with the host cell, these mutant parasites show lower infection levels. This reduction in infectivity is consistent with the opposing roles of the metacyclic-stage surface glycoproteins gp82 and gp90, which are key determinants of host cell invasion. Gp82 is a well-characterized adhesion molecule that actively promotes parasite entry by triggering calcium-dependent signaling pathways in both parasite and host cells, whereas gp90 acts as a negative regulator of infectivity, with higher surface expression correlating with reduced invasion efficiency, and does not induce Ca²^+^ mobilization (Ramirez et al., 1993; Málaga and Yoshida, 2001a). Together, these findings indicate that the TcVps34–Vps15 complex not only regulates autophagy and differentiation but also influences parasite establishment of infection. A deep understanding of these interactions not only will enhances the knowledge about *T. cruzi* biology but also opens opportunities for the discovery of new therapeutic targets against Chagas disease.

## Material and methods

### Cell cultures and transgenic parasites

*Trypanosoma cruzi* epimastigote forms (Y strain) were cultured at 28 °C for 7 days in LIT medium [5 g/L liver infusion, 5 g/L bacto-tryptose, 68 mM NaCl, 5.3 mM KCl, 22 mM Na_2_PO_4_, 0.2% (W/V) glucose, 0.002% (W/V) hemin] supplemented with 10% (V/V) newborn calf serum, 100,000 units/L penicillin and 100 mg/L streptomycin. Cell viability was assessed by direct microscopic examination, considering as viable those parasites that displayed active motility and preserved morphology characteristic of the stage being evaluated.

For stable transgenic parasite lines, the TcVps34 or TcVps15 gene constructs subcloned into the pRIBOTEX vector and tagged with HA at their C terminus (TcVps34-OE and TcVps15-OE, respectively) were used, as previously described (Schoijet et al., 2017). Then, parasites of Y strain were transfected with both constructs independently.

### *T*. *cruzi* differentiation protocol

*T*. *cruzi* metacyclogenesis induction was performed *in vitro* as in the previously published protocol (Vanrell et al., 2017). Briefly, epimastigotes of *T*. *cruzi* Y strain (or the overexpressing parasites TcVps34 and TcVps15) grown to stationary phase (5 x 10^7^ cells/mL) were collected by centrifugation at 2000 xg for 15 min, and resuspended at 5 x 10^8^ cells/mL in TAU medium (190 mM NaCl, 17 mM KCl, 2 mM MgCl_2_, 2 mM CaCl_2_, 0.035% sodium bicarbonate, 8 mM phosphate, pH 6.9). After 2 h at 37 °C (1st stage of metacyclogenesis), parasite samples were processed for microscopy or molecular studies. Similar procedures were conducted in control parasites maintained in LIT medium at 28 °C. In other cases, to complete the differentiation process, parasites were diluted 100 times in TAU 3AAG (TAU medium supplemented with 10 mM L-proline, 50 mM sodium L-glutamate, 2 mM sodium L-aspartate, and 10 mM glucose) or control media and maintained at 28 °C for 48 h (2nd stage of metacyclogenesis). The intermediate forms are those that remain attached to the flasks and metacyclic trypomastigotes remain in the supernatant. In some experiments, TAU medium was supplemented with 100 nM wortmannin (Sigma-Aldrich) as an autophagy inhibitor. For autophagy induction, 100 ng/μL rapamycin (Rap, LC Laboratories) was added to control media.

### Complement-mediated lysis susceptibility assay

To assess the resistance of differentiated parasites to complement-mediated lysis, an evaluation based on exposure of the parasite populations to fresh human serum (FHS) was performed. Parasite samples obtained after the metacyclogenesis protocol were combined with an equal volume of FHS and were incubated for 15 minutes at 37 °C. Epimastigotes are susceptible to complement system proteins present in the serum, whereas trypomastigotes are not. After the treatment, surviving parasites were quantified in a hemocytometer chamber using optical microscopy, based on their morphological integrity and motility. The resistance rate (%) was calculated as the number of trypomastigotes/total number of parasites counted on TAU 3AAG x 100.

### Monodansylcadaverine labeling assay

The assay was performed according to Munafó and Colombo (Munafó and Colombo, 2001). Briefly, after autophagy induction by serum deprivation, parasites were incubated with 0.05 mM MDC in PBS at 28 °C for 16 h. After incubation, cells were washed three times with PBS and immediately analyzed in an Olympus BX41 fluorescence microscope. To quantify the labeling of MDC, cells were washed twice in PBS and lysed in 10 mM Tris–HCl pH 8.0, containing 0.1% Triton X-100. MDC stain was measured by fluorescent photometry (excitation wavelength 380 nm, emission filter 525 nm). To normalize the measurements, the fluorescence of total DNA was quantified previously by staining with 0.2 μM ethidium bromide (excitation wavelength of 530 nm and an emission filter of 590 nm). Results were expressed as specific activity (percentage respect to the control).

### DQ-BSA labeling

The method was similar to that of the previous section, with the addition of 10 μg/mL dequenched BSA instead of MDC. Red DQ-BSA requires enzymatic cleavage in acidic intracellular lysosomal compartments to generate a highly fluorescent product that can be monitored by microscopy or flow cytometry. Parasites were subjected to the first period of metacyclogenesis, and 30 min earlier were incubated with DQ-BSA (10 μg/mL), then washed twice with PBS, fixed with 4% formaldehyde, and analyzed by microscopy. For flow cytometry the same treatment was used, but with no fixation. Cells were analyzed using a FACSCalibur flow cytometer (BD Biosciences) and FlowJo software.

### Indirect immunofluorescence and flow cytometry assays

For immunofluorescence, cells were fixed with 4% (W/V) paraformaldehyde in PBS for 15 min. Next, the cells were washed twice in Dulbecco’s PBS, pH 7.2, adhered to poly-L-lysine-coated coverslips, and permeabilized for 2 min with 0.3% Triton X-100. Cells were incubated for 10 min with 50 mM ammonium chloride, and washed again with PBS. Afterwards, they were blocked for 20 min with 3% bovine serum albumin in PBS, pH 8.0, and incubated for 1 h with rat anti-HA high-affinity monoclonal antibodies (Roche Applied Science) at 1:500 and rabbit anti-ATG8.1 polyclonal antibody at 1:700, kindly gifted by Dr. Vanina Alvarez (Alvarez et al., 2008). Cells were then washed in 3% bovine serum albumin, incubated with secondary antibodies anti-rabbit Alexa 488 at 1:500 and anti-rat Alexa 546 conjugate at 1:500 containing 5 mg/mL DAPI, and mounted with Vectashield (Vector Laboratories). Cells were observed in an Olympus BX41 fluorescence microscope. The number of autophagosomes was calculated using ImageJ software as the number of punctate-positive cells divided by the total number of cells in the same field. More than 100 cells were scored for each treatment. Data represent the means of three independent experiments. For the metacyclogenesis evaluation assays, the same procedure was followed using the monoclonal antibodies 3F6 and 1G7 at a 1:250 dilution, which label the surface glycoproteins Gp82 and Gp90, respectively. These antibodies were generously donated by Dr. Ana Claudia Torrecilhas, Federal University of São Paulo (UNIFESP).

For flow cytometry with surface molecules gp82 or gp90, live metacyclic trypomastigotes (1×10^7^) were incubated on ice for 1 h with monoclonal antibody 3 F6 or 1G7 at a 1:100 dilution, directed respectively to the proteins mentioned above. Then, the parasites were fixed with 4% paraformaldehyde for 20 min. Following washings in PBS, the parasites were incubated with Alexa Fluor 647-conjugated anti-IgG for 1 h at room temperature and the number of fluorescent parasites was estimated using a FACSCalibur flow cytometer (BD Biosciences) and FlowJo software.

### Western blot assays

For *T. cruzi* extracts, 2.5 - 5 × 10^7^ parasites previously incubated in LIT medium or differentiated in TAU and TAU 3AAG media were harvested by centrifugation at 1,500 g for 10 min and washed with phosphate-buffered saline (PBS). Cell pellets were then resuspended in lysis buffer (50 mM Tris-HCl buffer, pH 7.5, 0.5 mM phenylmethylsulfonyl fluoride and 14 mM 2-mercaptoethanol), and lysed by sonication.

Samples were then boiled and solved in 8% SDS-polyacrylamide gel electrophoresis. Afterward, proteins were transferred to nitrocellulose membranes, blocked with 5% non-fat milk for 1 h at room temperature and incubated ON at 4 °C with monoclonal antibody 3F6 or 1G7 against GP82 and GP90, respectively, at 1:1000 dilutions. Membranes were washed three times for 5 min each with phosphate-buffered saline (PBS) containing 0.05% Tween 20, incubated with anti-mouse IgG coupled to horseradish peroxidase (Sigma) for 1 h at RT, washed again under the same conditions and visualized by chemiluminescence. Densitometric analysis was performed using ImageJ software.

### Infection assays

Y wild-type and overexpressing epimastigote forms were differentiated to metacyclic trypomastigotes in a biphasic medium supplemented with human blood (Rodríguez Durán et al., 2021). Once the trypomastigotes were obtained, 24-well plates with Vero cells (2.5 - 5x10^4^ cells) were infected at a 15:1 ratio of parasites to host cells. Infections were allowed to proceed for 3 h, after which cultures were thoroughly washed with PBS to remove non-internalized parasites. The infection was then allowed to progress, and cells were fixed and stained at 48 h post-infection using the May-Grünwald Giemsa technique.

### Automated image-based analysis of *T. cruzi* infection

Infection levels were quantified using a custom image analysis pipeline. Briefly, infections were performed as described above, and infected cell cultures were fixed and stained with DAPI to visualize host cell nuclei and *T. cruzi* amastigote nuclei and kinetoplasts. Digital images were acquired under standardized conditions using an Olympus BX41 widefield fluorescence microscope equipped with an Olympus DP71 camera, and quantification was performed at 3, 24, and 48 h post-infection. We refined a pre-trained neural network with manually annotated images to accurately identify and classify infected cells, as well as to detect and count intracellular amastigotes. The model output provided both the percentage of infected cells and the mean number of amastigotes per infected cell. The algorithm was previously validated against manually annotated datasets to ensure accuracy and reproducibility. The output was verified using a graphical summary for each image. All quantifications were performed on images obtained from three independent experiments, for a total of 4472 observed host cells.

### Statistical analysis

Data were expressed as mean ± SEM of three independent experiments.

Differences were considered statistically significant at least when p < 0.05.

Significance levels for comparisons between two groups were determined with t test (2 tail). *= p < 0.05; **= p < 0.01; ***= p < 0.001.

Significance levels for comparisons between more than two groups were determined with one-way ANOVA and multi-comparison analysis was performed with *post hoc* Tukey’s test *= p < 0.05; **= p < 0.01; ***= p < 0.001.

Excel and GraphPad Prism 5 software were used for statistical analysis and the generation of graphs. See details in the figure legends.

To analyze the infection parameters, we fit a Generalized Linear Mixed Model (GLMM). Because this variable was non-normally distributed count data, we specified a negative binomial distribution with a log-link function. To compensate for the zero-inflation in our data, we set the zi-formula to 1. A similar approach was used for modelling the proportion of infected cells, specifying a binary response (0 = uninfected and 1 = infected) with a binomial distribution, using a logit-link function. Both models included the time post-infection (3, 24, and 48 hours) and the parasite line (WT, TcVps34-OE and TcVps15-OE) as fixed effects variables, along with their two-way interaction. The lack of independence between fields obtained from each of the 3 independent replicates, as well as the dependence between cells obtained from the same microscopic fields, were included as nested random intercepts. The models were fitted in R (4.3.3) using the glmmTMB package. Simple effects comparisons between the parasite lines at the different time points were carried out with the emmeans package.

## Results

### Overexpression of TcVps34 and TcVps15 induces metacyclogenesis

**Figure 1:**
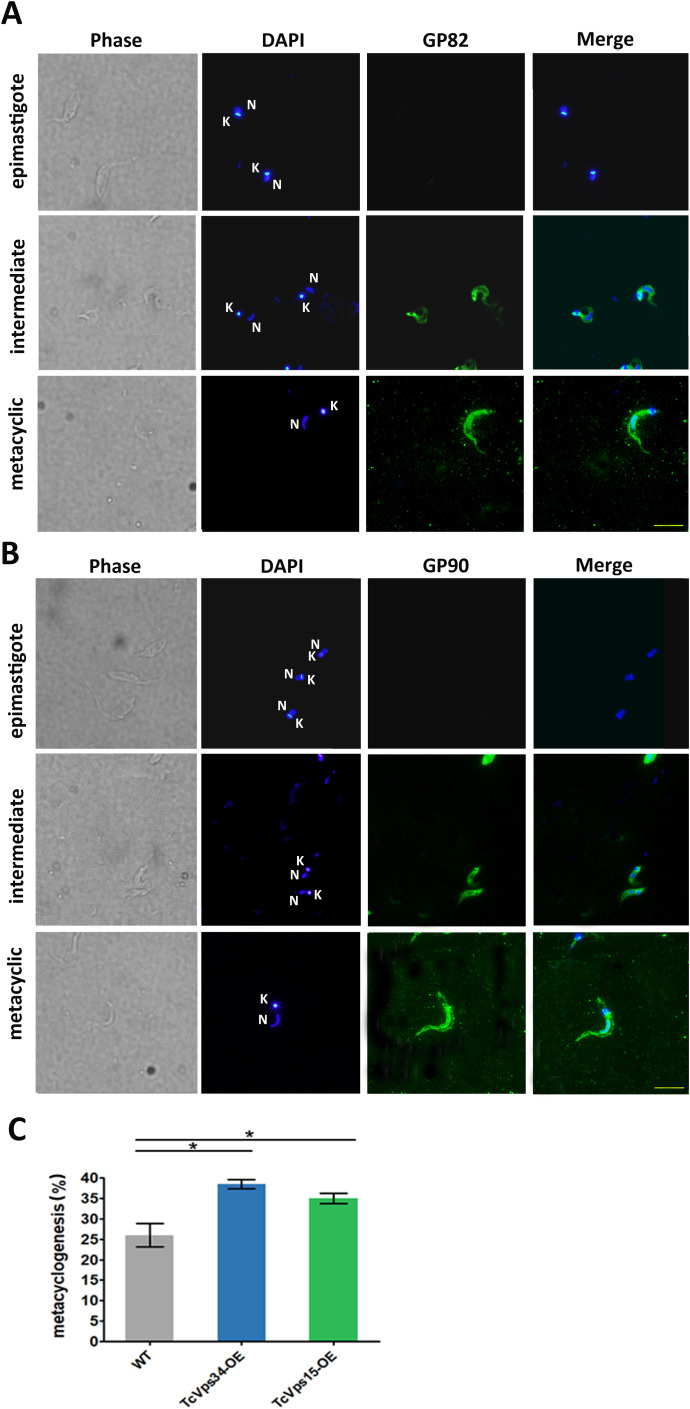
Effect of TcVps34 and TcVps15 overexpression on *in vitro* metacyclogenesis. Immunofluorescence was performed using exponentially growing epimastigotes or after 48 h of differentiation in TAU 3AAG medium. Gp82 and Gp90 proteins were detected using the monoclonal antibodies 3F6 **(A)** or 1G7 **(B)** and incubated with secondary antibody Alexa Fluor 488. DAPI was used to stain the nucleus (N) and kinetoplast (K). Left panels correspond to the bright field images. Scale bar = 10 μm. **(C)** The percentage of metacyclic trypomastigotes was quantified by staining with DAPI. Numbers are derived from three independent experiments where 300 cells were analyzed. Results are means ± SEM. *P < 0.05 (unpaired two-tailed Student’s t test).

Previous work has shown that the autophagy process is stimulated during metacyclogenesis in *T. cruzi* (Vanrell et al., 2017). Since our group has shown that overexpression of TcVps34 and TcVps15 in *T. cruzi* epimastigotes stimulates autophagy, we decided to investigate whether the differentiation process is also stimulated in these overexpressing parasites. For this purpose, strain Y parasites that overexpress these enzymes separately (TcVps34-OE and TcVps15-OE) were differentiated first in TAU and then in TAU 3AAG medium for 48 h (2nd stage of metacyclogenesis). Differentiation of epimastigotes to metacyclic trypomastigotes was quantified by DAPI staining. In addition, staining with antibodies for the surface glycoproteins Gp82 and Gp90, expressed on metacyclic trypomastigotes, was performed. As can be seen in **Figures 1A, B**, both proteins are located in the plasma membrane in metacyclic trypomastigotes. In intermediate forms, both Gp82 and Gp90 presented a dual localization, in the plasma membrane and in intracellular compartments, and they are not expressed in epimastigotes. After quantification, it could be observed that metacyclogenesis was significantly increased in both overexpressing parasites compared to wild-type parasites (**Figure 1C**). The resistance of the parasites to complement lysis was also evaluated after their differentiation in TAU 3AAG medium. Since transgenic parasites showed no significant differences with respect to wild-type ones (**Supplementary Figure 1**), we hypothesized that the obtained metacyclic trypomastigotes from the overexpressing cell lines are less resistant to the complement-mediated lysis.

### Autophagy in differentiated TcVps34-OE and TcVps15-OE parasites

**Figure 2:**
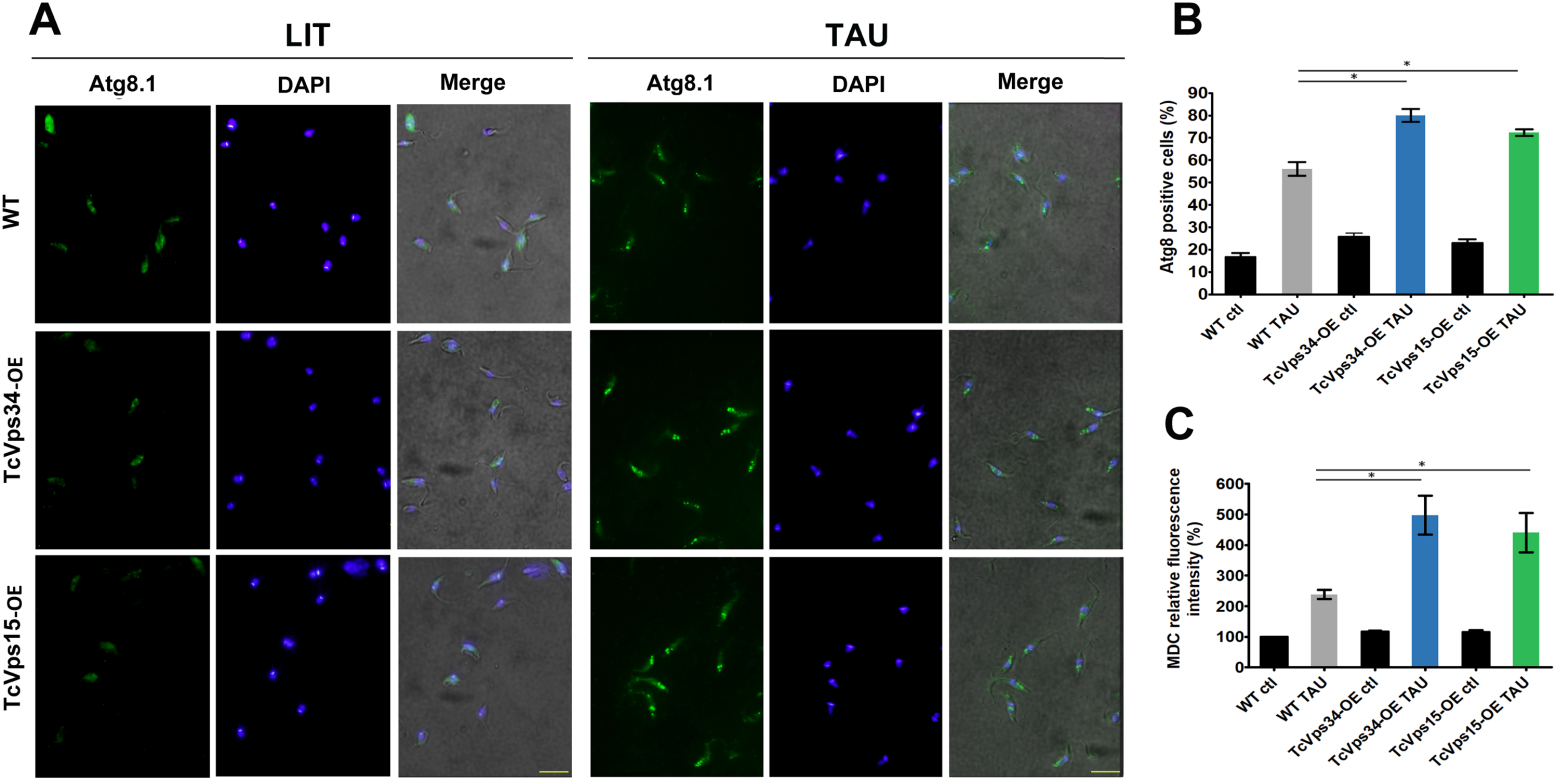
Autophagy is increased in differentiated parasites that overexpress TcVps34 and TcVps15. Wild-type (WT), TcVps34-OE, and TcVps15-OE epimastigotes were incubated under control (LIT medium at 28 °C) or differentiation conditions (TAU medium at 37 °C) for 2 h to induce differentiation, and then autophagy was measured by Atg8.1 and MDC labelling. **(A)** Detection of the TcAtg8.1 protein by immunofluorescence. Scale bar: 10 μm. **(B)** Percentage of parasites with more than two Atg8.1-positive vesicles under each condition. Autophagy response was analyzed in each condition by quantification of the percentage of parasites with more than two TcAtg8.1-positive vesicles. Number of counted cells: 200. Error bars represent the standard error of the mean (SEM) (t-test, p <0.05, asterisks). **(C)** Labelling with MDC. Differentiated parasites were treated as described in materials and methods and intracellular MDC was measured by fluorescence photometry. The data represent the mean SEM of three independent experiments (p <0.05, *).

The induction of autophagy has been reported to promote metacyclogenesis (Vanrell et al., 2017). To investigate the biological relevance of TcVps34 and TcVps15 in this process, we subjected TcVps34-OE and TcVps15-OE epimastigote cells to the first period of differentiation and evaluated autophagy by immunofluorescence microscopy using antibodies against the autophagy marker Atg8.1. Then, we quantified the number of parasites with more than two Atg8.1-positive vesicles (**Figures 2A, B**). Overexpressing parasites showed an increase in the number of parasites with more than two Atg8.1-positive vesicles compared to the control in TAU medium (1.429 ± 0.036 fold-increase for TcVps34-OE and 1.292 ± 0.051 for TcVps15-OE cells, compared to wild-type cells). The levels of autophagy were also quantified using monodansylcadaverine (MDC), a specific *in vivo* marker for autophagic vacuoles. As shown in **Figure 2C**, both TcVps34-OE and TcVps15-OE epimastigotes subjected to nutritional stress in TAU differentiation medium displayed a higher frequency of MDC labeling compared to wild-type cells.

**Figure 3:**
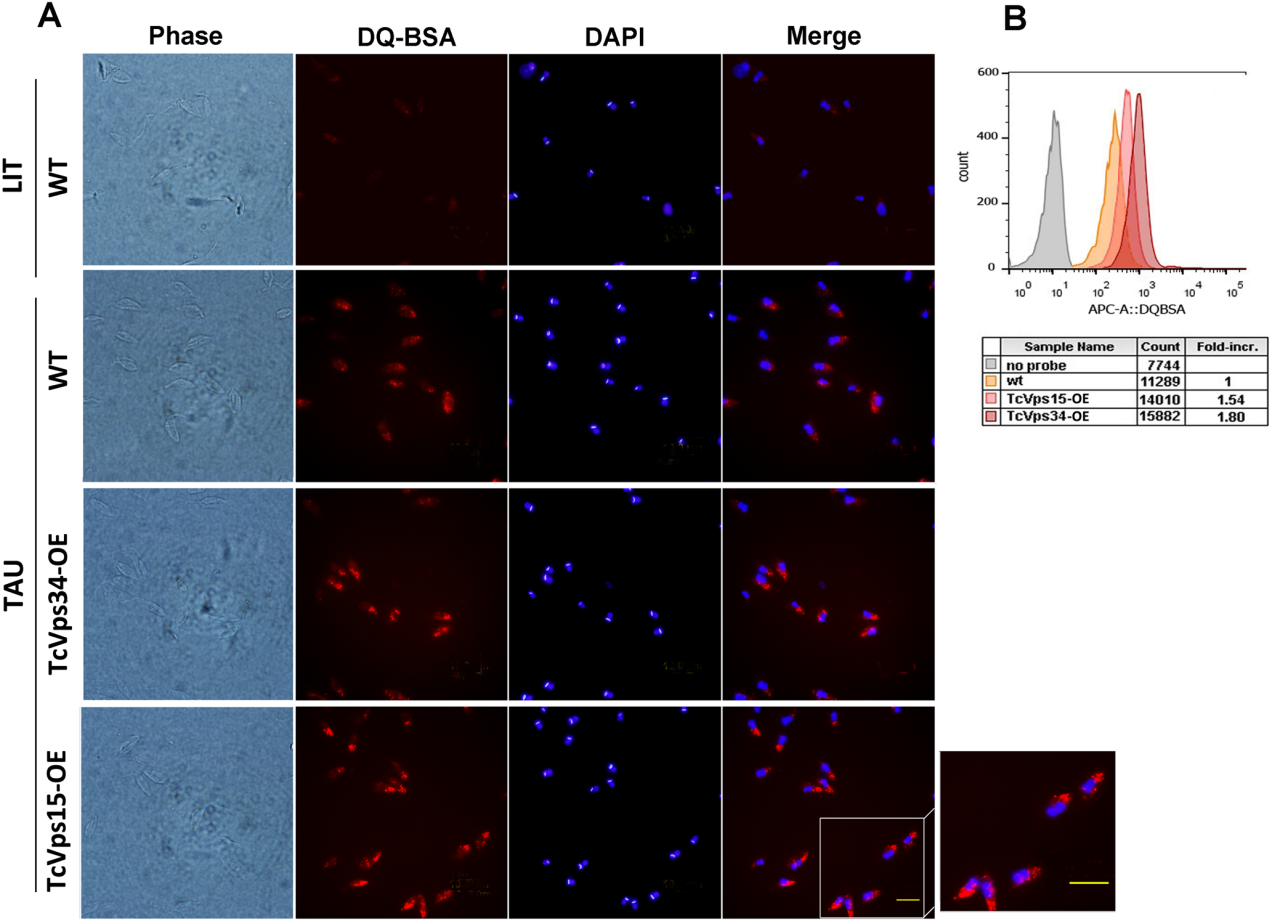
Evaluation of hydrolytic compartments in TcVps34-OE and TcVps15-OE parasites. **(A)** Immunofluorescence analysis of DQ-BSA distribution in wild-type epimastigotes non-induced (LIT-top panel) or wild-type, TcVps34-OE and TcVps15-OE parasites induced to undergo autophagy (TAU) during the first stage of metacyclogenesis. Scale bar: 10 μm. **(B)** DQ-BSA fluorescence intensities were quantified by flow cytometry. Data represent the mean of three independent experiments and were analyzed by FlowJo software.

In addition to the increase in autophagosomes number, the activation of autophagy in mammalian cells is also marked by a rise in the number of lysosomes and autolysosomes necessary for the lysis of engulfed materials (Vázquez and Colombo, 2009). To assess this in our model, we utilized self-quenched albumin (DQ-BSA), which serves as an indicator for hydrolytic compartments. As illustrated in **Figure 3**, TcVps34-OE and TcVps15-OE epimastigotes exposed to nutritional stress in TAU differentiation medium displayed a marked increase in the number of lysosomes compared to the wild-type group. Taken together, these results evidence that TcVps34-OE and TcVps15-OE parasites show an increased autophagic activity after two hours of metacyclogenesis induction.

### Effect of autophagy modulators on differentiated TcVps34-OE and TcVps15-OE parasites

**Figure 4:**
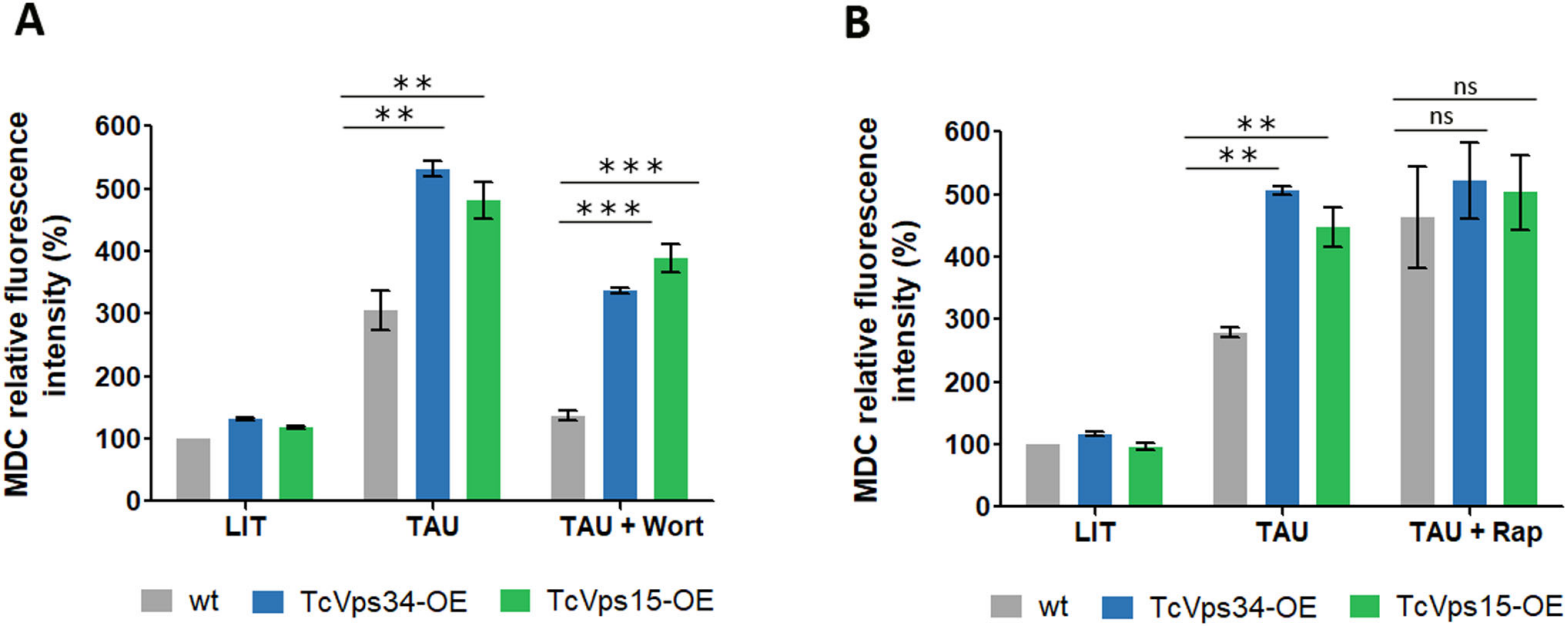
Effect of autophagy inhibitors and inducers on TcVp34-OE and TcVps15-OE parasites. Y strain *T. cruzi* wild-type, TcVp34 and TcVps15 overexpressing parasites were incubated in LIT, or subjected to differentiation for two hours in TAU medium at 37 °C in the absence or presence of 100 nM wortmannin (Wort) **(A)**, and 100 ng/μL rapamycin (Rap) **(B)**. After these treatments, parasites were processed to detect autophagosomes using MDC staining. The percentage of parasites labeled with these markers under each condition was quantified. Data shown represent the mean ± SEM from three independent experiments. **p < 0.01, ***p < 0.001 (Tukey’s test).

Next, we evaluated the effect of two autophagy-modulating drugs, the PI3K inhibitor wortmannin and the autophagy inducer rapamycin. As seen in **Figure 4A**, treatment with wortmannin (Wort) during differentiation induction in TAU medium for 2 h (1st stage of metacyclogenesis) decreases the autophagy process, as determined by measuring MDC after treatment, with this effect being significantly lower in parasites overexpressing TcVps34 and TcVps15. On the other hand, treatment with rapamycin (Rap) during induction of differentiation as described above induces an increase in the number of autophagic vesicles, although this effect was not statistically significant in parasites overexpressing TcVps34 and TcVps15 compared to wild-type parasites (**Figure 4B**).

### Effect of overexpression of TcVps34 and TcVps15 on host cell infection

**Figure 5:**
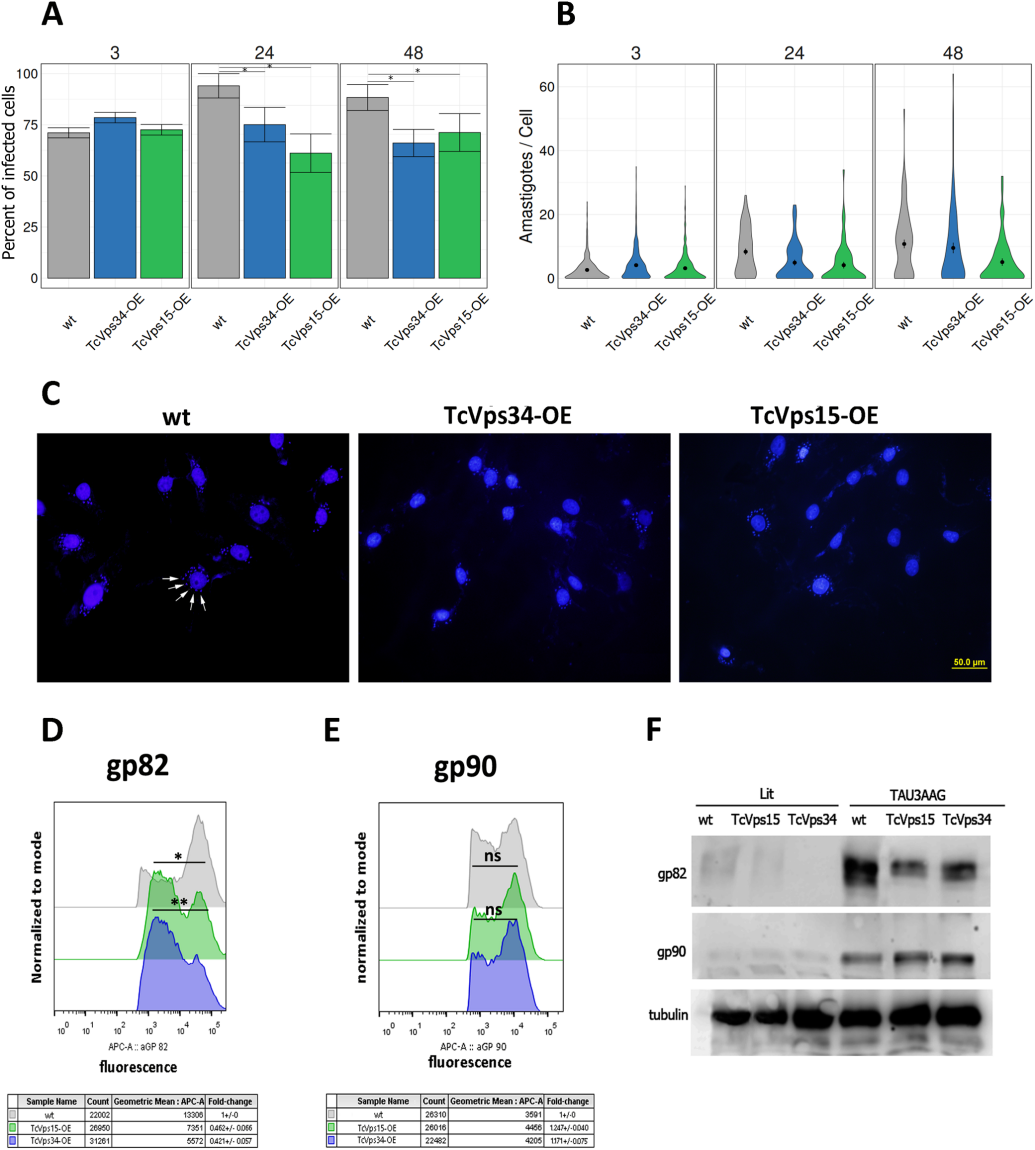
Effects of overexpression of TcVp34 and TcVps15 proteins in the invasion of mammalian cells. Parasites were allowed to infect Vero cells for 3 h, after which cultures were washed with PBS to remove non-internalized parasites. Quantification of infection was then performed at 3, 24, and 48 h post-infection. There was a significant difference in the percentage of infected Vero cells at 24 and 48 h **(A)** but not in the number of intracellular amastigotes per infected cell observed 48 h post infection **(B)**. Values are means ± SD (n = 3), ns, no significant differences, and *P < 0.05 by Student’s t-test. **(C)** Representative DAPI stained images of Vero cells infected. The white arrows indicate *T. cruzi* amastigotes. **(D, E)** Surface expression levels of gp82 and gp90 in wild-type metacyclic trypomastigotes or overexpressing TcVps34 and TcVps15. The graphs show flow cytometry analysis after incubation with primary antibodies 3F6 and 1G7, and detection with a fluorophore-conjugated secondary antibody. Fluorescence intensity analysis revealed a significant reduction in surface gp82 levels in TcVps15 and TcVps34-overexpressing parasites, whereas gp90 expression remained statistically unchanged. Data shown represent the mean ± SEM from three independent experiments. *p < 0.1, **p < 0.01 (paired t-test). **(F)** Protein extracts from 2.5 - 5 × 10^7^ parasites were obtained from wild-type (WT), TcVps34-OE, and TcVps15-OE lines previously incubated under control conditions in LIT medium or differentiated by incubation in TAU followed by TAU 3AAG medium. Samples were subjected to electrophoresis in a 8% polyacrylamide gel, transferred to nitrocellulose membranes, and analyzed by Western blot using the monoclonal antibodies 3F6 (anti-gp82) and 1G7 (anti-gp90) at a 1:1000 dilution. Bands of approximately 82 kDa and 90 kDa, corresponding to gp82 and gp90, respectively, were detected in differentiated parasites. The 50 kDa band corresponds to tubulin, used as a loading control. Densitometric analysis was performed using ImageJ software.

To study the role of increased autophagy and metacyclogenesis in the transgenic parasites TcVps34 and TcVps15 in modulating host cell infection, we evaluated the infection parameters with these transgenic parasites at 3, 24, and 48 h post-infection. **Figure 5A** shows that although there were no significant differences at 3 h post-infection, a significantly infection reduction was observed in TcVps34-OE and TcVps15-OE parasites at 24 and 48 h post-infection, while the replication of intracellular amastigotes was not significantly affected by the overexpression of these proteins (**Figure 5B**). These results were quantified by May-Grünwald Giemsa staining and alternatively, DAPI was used to visualize the DNA content in parasites and Vero cells, as shown in representative **Figure 5C**. Since previous reports from our group and others have shown that TcVps34 is involved in endocytosis and intracellular membrane trafficking (Schoijet et al., 2008; Nascimbeni et al., 2017), we subsequently evaluated the expression of the surface glycoproteins gp90 and gp82, which act as inhibitor and promoter of target cell invasion respectively (Ramirez et al., 1993; Málaga and Yoshida, 2001), by flow cytometry in these transgenic parasites. Both groups of overexpressing parasites showed statistically significant reduction levels of gp82 expression (**Figure 5D**), while in the case of gp90, although a slight increase in the positive signal for this marker was observed in the transgenic parasites, these levels were not significantly higher than in the control (**Figure 5E**). To further evaluate whether the differences observed by flow cytometry were reflected at the total protein level, we performed Western blot analysis using extracts from WT, TcVps34-OE, and TcVps15-OE parasites maintained in LIT medium or differentiated in TAU followed by TAU 3AAG. As shown in **Figure 5F**, gp82 and gp90 were detected as bands of approximately 82 kDa and 90 kDa, respectively, predominantly in differentiated parasites, whereas their expression was negligible in epimastigotes maintained in LIT medium. Consistent with the cytometry data, differentiated TcVps34-OE and TcVps15-OE parasites displayed reduced gp82 protein levels compared to WT, with fold change values of 0,406 **±** 0,036 and 0,318 **±** 0,027, respectively. On the other hand, gp90 levels showed a more modest variation, with fold changes of 1,690 **±** 0,140 in TcVps34-OE and 1,434 **±** 0,043 in TcVps15-OE parasites. In summary, vesicular trafficking alterations resulting from TcVps34 or TcVps15 overexpression would affect not only the delivery and surface distribution of infection-related glycoproteins but also their overall protein levels, which together may compromise the parasite’s ability to establish an infection.

## Discussion

Autophagy and metacyclogenesis are two critical processes in the life cycle of *Trypanosoma cruzi*, and understanding their interrelationship will help to clarify how this parasite adapts and survives in different environments, especially when transitioning between its various life stages. In this work, our findings highlighted the pivotal roles of the PI3K TcVps34 and its regulatory partner TcVps15 in these processes. Our results demonstrate that overexpression of these proteins not only enhances the autophagic pathway but also significantly promotes the differentiation of epimastigotes into metacyclic trypomastigotes. This suggests a coordinated regulatory mechanism where autophagy acts as a facilitator for the transition between life stages of the parasite, enabling it to adapt to environmental stresses encountered during its life cycle.

In our studies, we specifically observed a marked elevation in the number of parasites exhibiting multiple autophagic vesicles in TcVps34-OE and TcVps15-OE cells compared with wild-type controls after two hours of metacyclogenesis induction, as evidenced by TcAtg8.1 labeling and MDC staining. This aligns with previous literature that links autophagy to the differentiation process in *T. cruzi*, highlighting the potential for autophagy to facilitate the transition to infective forms (Vanrell et al., 2017). In addition, the analysis of hydrolytic compartments using DQ-BSA revealed that the overexpressing parasites had enhanced lysosomal activity, further supporting the notion that autophagy is actively engaged during metacyclogenesis. The utilization of pharmacological agents such as wortmannin and rapamycin contributed to elucidating the dynamics of autophagy regulation in this parasite, confirming that inhibition of PI3K can dampen the autophagic response while induction through rapamycin enhances it, albeit with varying effectiveness in these parasites. Specifically, wortmannin decreases the autophagic response when introduced during the differentiation induction in TAU medium. This reduction in autophagy negatively impacts the metacyclogenesis process, suggesting once more that autophagy is crucial for this differentiation. Conversely, while rapamycin enhanced autophagic activity, the lack of a significant increase in differentiation suggests that there may be a threshold of autophagic activity necessary for optimal differentiation, beyond which further stimulation does not confer additional benefits. This may also be due to that TOR activity is assumed to be regulated via various feedback loops; for example, in *T. brucei*, rapamycin induced the formation of a high number of autophagosomes by TORC2 inhibition but did not inhibit TORC1 (Barquilla et al., 2008; Menna-Barreto, 2019). Interestingly, the TcVps34-OE and TcVps15-OE parasites exhibited reduced infection rates in Vero cells. This observation raises intriguing questions about the balance between metacyclogenesis and infection capability. It suggests that while these transgenic parasites are more adept at entering the metacyclic stage, the heightened autophagic process may alter their interaction with host cells, possibly enhancing immune evasion or modifying the signaling pathways required for the proper establishment of infection. This effect prompts further investigation to better understand the implications of elevated autophagic processes on parasite-host interactions. In line with this idea, it is worth noting that although metacyclogenesis was increased in these transgenic parasites, they did not exhibit higher resistance to complement-mediated lysis after incubation with FHS. This finding aligns with their reduced infectivity, since virulent forms of *T. cruzi* are generally more resistant to the mammalian complement system (Kierszenbaum et al., 1976; Cestari and Ramirez, 2010). This observation suggests that while TcVps34 and TcVps15 play key roles in regulating autophagy and differentiation, they may not directly modulate the expression or activity of complement-evasion molecules, such as complement regulatory proteins. Instead, these alterations may arise from defects in vesicular trafficking within the transgenic parasites, potentially disrupting membrane organization and indirectly affecting the surface distribution of key glycoproteins such as gp82 and gp90. Consistent with this view, our cytometry data align with a recent study that reported comparable glycoprotein imbalances following TcVps34 overexpression in *T. cruzi* G strain (Nájera et al., 2024). Although gp82 and gp90 are both GPI-anchored surface molecules expressed in metacyclic trypomastigotes, previous studies have demonstrated that they follow distinct intracellular trafficking pathways during metacyclogenesis. Gp90 is transported through the classical secretory route and rapidly delivered to the parasite surface via the flagellar pocket, whereas gp82 is transiently retained in lysosome-related organelles, where it colocalizes with cruzipain, before being redirected to the plasma membrane at later stages of differentiation (Bayer-Santos et al., 2013). In this context, alterations in vesicular trafficking associated with TcVps34 or TcVps15 overexpression are expected to preferentially impact gp82, whose trafficking depends on endosomal and lysosome-related compartments regulated by the Vps34/Vps15 complex, while having a more limited effect on gp90. Consistently, Western blot analysis showed a more pronounced reduction in total gp82 levels compared to gp90, suggesting that gp82 synthesis and/or stability might be more affected in the overexpressing parasites. These defects likely contribute to the reduced infectivity observed in the transgenic lines. Together, these findings suggest that vesicular transport defects resulting from altered expression of Vps components may contribute to the downregulation of virulence factors on the surface of infective forms. In addition, it is important to highlight that the transgenic parasites do not seem to exhibit a major defect in the initial steps of host cell entry (**Figure 5A**, 3h). However, once inside the host cell, their ability to successfully establish the infection appears to be negatively affected (**Figure 5A**, 24h and 48h). Several factors could contribute to the impairment of establishing an infection, including a reduced efficiency in escaping from the parasitophorous vacuole or a higher susceptibility to lysosomal degradation during the early intracellular stages. These possibilities are compatible with the idea that altered vesicular dynamics in the TcVps34-OE and TcVps15-OE lines may interfere with the post-entry processes required to establish a productive infection. Notably, once infection is successfully established, intracellular replication seems to proceed normally, as indicated by the similar number of amastigotes per cell in wild-type and transgenic parasites (**Figure 5E**). This observation further supports the notion that the primary defect in these transgenic cell lines lies in the events following entry rather than in the replicative capacity of amastigotes. Future experiments focusing for example, on the mechanisms governing vacuolar escape and early intracellular survival will help clarify the basis of these defects and further elucidate how alterations in Vps-mediated trafficking shape the interplay between autophagy, differentiation, and infection in *T. cruzi*.

In addition, the reservosomal content consumed during metacyclogenesis and the presence of Atg8 in this organelle strongly suggest that there is a crosstalk between autophagy and reservosomes. Moreover, parasite starvation promotes the delivery of Cruzipain, an important virulence factor in *T. cruzi*, to the reservosomes (Losinno et al., 2021). In view of this, the autophagic system connects with various cellular processes and affects the interactions between different pathogens and host cells. In infections caused by protozoa, the role of autophagy is a topic of debate, as research findings have produced conflicting evidence that often depends on the specific experimental model used. Some studies indicate that parasites may use autophagy to avoid defenses from host cells, while others find that the host employs autophagy to fight off the parasites (Aguilera et al., 2024). Despite these differing points of view, it is clear that the autophagic system plays a significant role in the development and severity of protozoan infections, making it a potential target for new drug development.

In summary, this study underscores the complex interplay between autophagy and metacyclogenesis in *T. cruzi*, revealing that while enhanced autophagic activity supports differentiation, the overexpression of TcVps34 and its interacting partner TcVps15 may also impose constraints on the parasite’s infectivity. Therefore, by modulating the autophagic response, it may be possible to influence not only the lifecycle of the parasite but also its ability to establish infections. Future research should focus on elucidating the molecular mechanisms underlying these interactions and exploring potential therapeutic interventions that exploit this relationship to effectively struggle with Chagas disease.

## Data Availability

The raw data supporting the conclusions of this article will be made available by the authors, upon request.

## Supplementary material

Supplementary Figure 1 Resistance of parasites to complement-mediated lysis. Wild-type (WT), TcVps34-OE, and TcVps15-OE epimastigotes differentiated in TAU 3AAG medium for 48 h were incubated with 50% FHS at 37 °C for 15 min and live parasites were counted. Values are means ± SD (n = 3), ns, no significant differences.
