# Supplementary material for: The TcVps34–TcVps15 complex regulates parasite metacyclogenesis and host cell infection in Trypanosoma cruzi
Source: Front Cell Infect Microbiol. 2026 Apr 21;16:1668322. doi: 10.3389/fcimb.2026.1668322 (PMC13139090; doi:10.3389/fcimb.2026.1668322)
Supplement: Supplementary Figure 1 — Resistance of parasites to complement-mediated lysis. Wild-type (WT), TcVps34-OE, and TcVps15-OE epimastigotes differentiated in TAU 3AAG medium for 48 h were incubated with 50% FHS at 37 °C for 15 min and live parasites were counted. Values are means ± SD (n = 3), ns, no significant differences. [file DataSheet1.pdf]

Supplementary figure 1

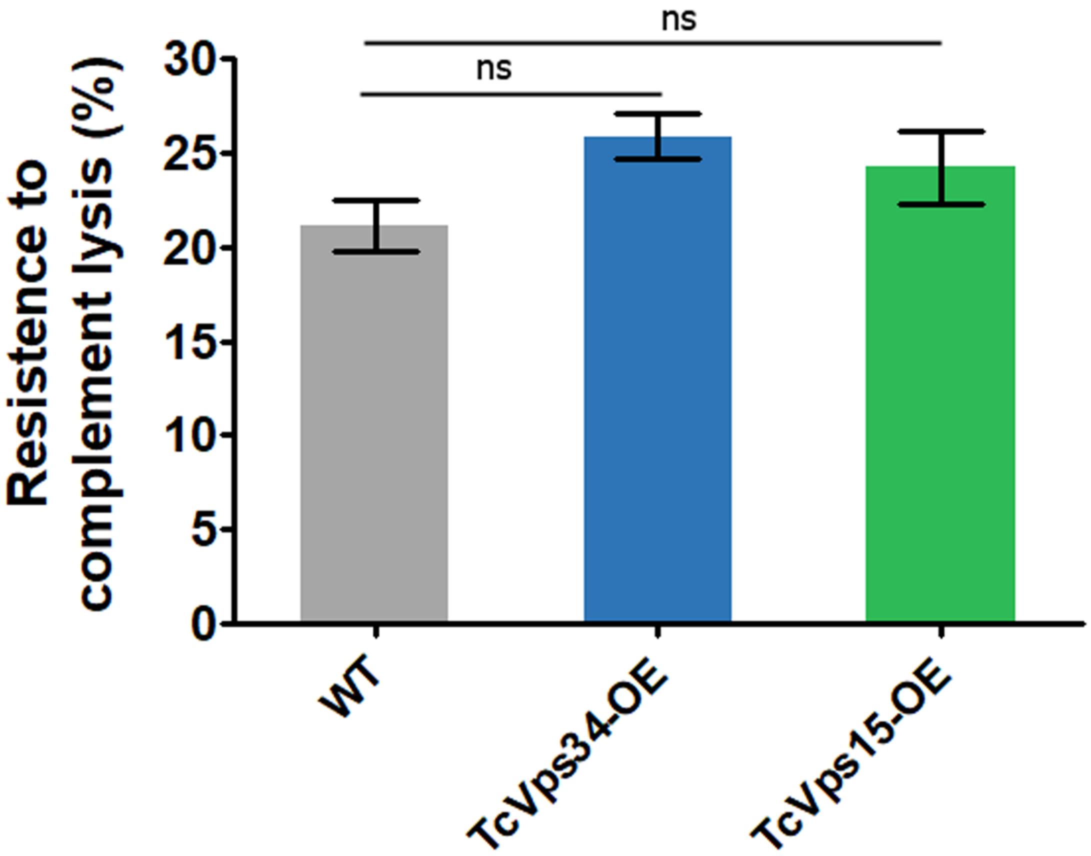

SUPPLEMENTARY FIGURE 1. Resistance of parasites to complement-mediated lysis. Wild-type (WT), TcVps34-OE, and TcVps15-OE epimastigotes differentiated in TAU 3AAG medium for 48 h were incubated with 50% FHS at 37 °C for 15 min and live parasites were counted. Values are means ± SD (n = 3), ns, no significant differences.
